# Secondary Structure of a Conserved Domain in the Intron of Influenza A NS1 mRNA

**DOI:** 10.1371/journal.pone.0070615

**Published:** 2013-09-02

**Authors:** Salvatore F. Priore, Elzbieta Kierzek, Ryszard Kierzek, Jayson R. Baman, Walter N. Moss, Lumbini I. Dela-Moss, Douglas H. Turner

**Affiliations:** 1 Department of Chemistry and Center for RNA Biology, University of Rochester, Rochester, New York, United States of America; 2 Institute of Bioorganic Chemistry, Polish Academy of Sciences, Poznan, Poland; Mount Sinai School of Medicine, United States of America

## Abstract

Influenza A virus is a segmented single-stranded (−)RNA virus that causes substantial annual morbidity and mortality. The transcriptome of influenza A is predicted to have extensive RNA secondary structure. The smallest genome segment, segment 8, encodes two proteins, NS1 and NEP, via alternative splicing. A conserved RNA domain in the intron of segment 8 may be important for regulating production of NS1. Two different multi-branch loop structures have been proposed for this region. A combination of *in vitro* chemical mapping and isoenergetic microarray techniques demonstrate that the consensus sequence for this region folds into a hairpin conformation. These results provide an alternative folding for this region and a foundation for designing experiments to probe its functional role in the influenza life cycle.

## Introduction

Influenza A is a segmented, negative-sense RNA virus that causes substantial morbidity and mortality on an annual basis. It is estimated that approximately 25,000 deaths result from influenza infections each year in the United States alone [1]. Influenza A also has the potential to form pandemic strains, which can have devastating public health implications. Because influenza virus uses RNA exclusively throughout its life-cycle, it may contain functionally important RNA secondary structures as observed in other RNA viruses [2]–[8]. A bioinformatics study revealed widespread predicted structure throughout the coding regions of influenza A, predominantly in the plus-sense (protein coding) orientation [9]. Folding stability is also predicted to be host-specific [10], [11]. Thus, secondary structure could also be an important consideration for host range.

**Figure 1:**
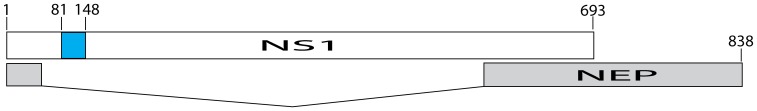
Diagram of the segment 8 (NS1/NEP) coding region. Numbering begins at the start of the NS1 open reading frame (ORF). White and shaded bars depict NS1 and NEP ORFs, respectively. Diagonal lines indicate the segment 8 intron and the blue box represents the 81–148 nt region.

The smallest segment of influenza A, segment 8 (NS1/NEP), codes for two proteins via alternative splicing (Figure 1) and is predicted to have wide-spread RNA structure [10]. In a study by Ilyinskii, et al. [12], a set of mutations disrupting the predicted RNA secondary structure of the intronic nucleotides 81–148 (numbering is from the start of the NS1 open reading frame) were linked to the down-regulation of NS1 protein. Despite this important result, no experimental RNA structure determination has been reported for this region. Ilyinskii, et al. proposed a 3-way multi-branch loop using an RNA secondary structure prediction algorithm called Mfold [12], [13]. Subsequently, a slightly different multi-branch loop structure was proposed [9]. Here, a hairpin model for the consensus sequence (see Materials and Methods) of the 81–148 nucleotide region is proposed on the basis of chemical mapping experiments and minimization of free energy (MFE) RNA secondary structure predictions [14], [15]. Coincidently, the consensus sequence for this region is identical to the 1918 pandemic strain, which led to the worst influenza A outbreak in history [16].

Knowledge of RNA secondary structure informs interrogation of function by facilitating design and interpretation of mutational studies. Once function is established, potential therapeutics against RNA structure can be designed. For example, databases that correlate small molecule binding patterns to RNA structural motifs can facilitate rapid identification of lead therapeutic compounds for a given RNA structure [17]. The hairpin reported here for nucleotides 81–148 of the NS1 intron should facilitate further functional analysis of this region.

## Results

### Comparison of Structural Models

**Figure 2:**
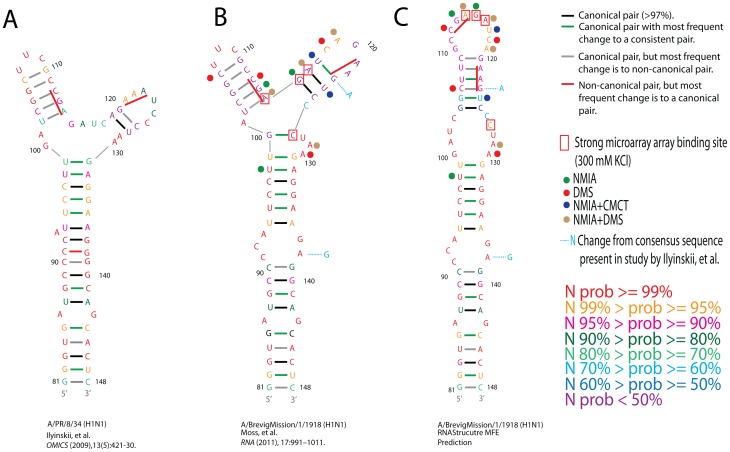
Structural models for nucleotides 81–148 of influenza A segment 8 (NS1/NEP). Numbering begins at the start of the NS1 open reading frame (ORF). Structural model A comes from Ilyinskii, et al. [12] and the sequence is from strain A/PR/8/34 (H1N1), B is modeled [9] with RNAalifold [18], and C is the MFE prediction from RNAstructure [14], [15]. B and C have the consensus sequence for this region based on an alignment of 1017 unique sequences and can be found in many strains, including A/BrevigMission/1918 (H1N1), which caused the most deadly pandemic in history [16]. RNAstructure free energy predictions at 37°C for sequences and structures A, B, and C are −7.1, −8.2, and −12.0 kcal/mol, respectively [15]. These free energy predictions exclude the GC pair at 100/127 in structure B, because it is predicted not to form on the basis of thermodynamic parameters for secondary structure [14]. Tertiary interactions may allow this pair to form or, alternatively, a C127/G131 base pair to form closing a UAA triloop. UAA triloops are common [42], [43] and can form tertiary interactions [24], [44], [45]. Individual nucleotides are colored based on probabilities from RNAstructure partition function calculations as shown in the key [19]. Colors of lines between nucleotides indicate type of conservation of pairing (see tables in Figure S1). Strong modification sites for NMIA, CMCT, and DMS are indicated by colored-dots next to each reactive nucleotide. Red boxes indicate the center nucleotide of strongly binding iso-energetic microarray probes in 10 mM Tris-HCl, pH 7.0, 300 mM KCl, and 10 mM MgCl_2_. Probe 127 could bind strongly to site 91, but it binds stronger than probe 91, indicating that site 127 is a true binding site for probe 127. Medium binding sites are not shown, but are centered at nucleotides 91 and 128. Light blue nucleotides next to structures B and C indicate positions in which sequence A differs from the consensus sequence. Solid red bars indicate the codons that were changed to GCG by Ilyinskii, et al. and led to down-regulation of NS1 protein [12]. The chemical mapping data, particularly the strong reactivity of nucleotides 112 and 113, are consistent with structure C but not with structure B.

Figure 2 shows three different predictions for the 81–148 nucleotide region of NS1 mRNA. Structure A is the model from Ilyinskii, et al. [12] based on the sequence of influenza strain A/PR/8/34 (H1N1). Structure B was predicted for the consensus sequence with RNAalifold [18] as part of a genome-wide survey for influenza A RNA secondary structures [9]. Structure C is the MFE prediction by RNAstructure [14], [15] for the consensus sequence. All three structures exhibit over 90% base pair conservation, largely because the stem below base pair 99/131 has high conservation in all three models (see Figure S1 for complete co-variation data). However, the region above base pair 94/136 of each structure is folded quite differently with free energy of folding for sequences and structures A, B, and C predicted by RNAstructure to be −7.1, −8.2, and −12.9 kcal/mol, respectively, at 37°C. Partition function calculations [19] provide the base pairing probabilities annotated on each structure. Structure A has two changes from the consensus sequence for this region. The G→A transition at nucleotide 123 disfavors prediction of structure C because Mfold and RNAstructure assign an unfavorable free energy to CA pairs, although they do form *in vivo* and *in vitro* [20]–[22]. Thus, sequence A has a much higher probability than the consensus sequence for the shared helix that begins at nucleotide 102 in structures A and B (see Figure 2). Because nucleotide 123 is a G in the consensus sequence, RNAstructure favors structure C and predicts low base pair probabilities for the multi-branch loop in structure B. Thus, based on MFE predictions structure C is expected to be the favored conformation for the consensus sequence at 37°C.

### Experimental Secondary Structure Determination

To better define the folding of this region, *in vitro* mapping experiments with small molecules (DMS, NMIA, and CMCT) and isoenergetic microarrays were performed on the consensus sequence as determined from all available unique influenza A strains. Small molecules probe the structure of folded RNA by modifying the base or sugar of unpaired, or weakly paired, nucleotides. Universal isoenergetic microarrays contain 861 different modified pentamer and hexamer oligonucleotides, thus providing complementarity to most sequences. The oligonucleotides are designed to bind with roughly equivalent stability to unpaired regions of a target RNA in order to minimize the effect of sequence dependence on binding. Folded RNA is hybridized to the array and binding to a probe reveals unpaired, or weakly paired, regions of the molecule. This technique is helpful for the design of potential therapeutics [23]. In summary, strong reactivity with small molecules or strong binding to microarray probes typically indicates unstructured (non-base paired) regions of an RNA structure.

The experimental results are overlaid on structural models B and C in Figure 2. The pattern of chemical reactivity and probe accessibility clearly favors structure C. Many reactive sites occur in predicted structured regions of model B, but are clustered in the hairpin loop of structure C. Specifically in support of structure C, positions 112 and 113 are reactive, but are inside a helix in structure B (Figures 2, S2, and S3). Positions 98, 106, and 124 are also reactive, although predicted to be double-stranded in both B and C. This is not inconsistent with either structure B or C, however, as chemical reactivity at or next to a G-U pair or at the end of helix is commonly observed [14]. Microarray binding sites only occur in either a hairpin or internal loop in structure C. (Table 1, Figures 2 and S4). Analysis of hybridization results in 10 mM MgCl_2_, 300 mM KCl showed that probes bind strongly to sites 114, 115, and 116 in the hairpin loop of structure C, whereas these sites are involved in base pairing in structure B. Interpretation of probes complementary to sites 127 (strong binding), 128 and 91(medium binding) is more complicated because they have potential alternative binding sites. However, sites 127 and128 are likely accessible because this region also shows chemical mapping reactivity (Figure 2). Interestingly, the hairpin loop of structure C also had strong or medium binding of five probes not completely Watson-Crick paired (i.e. forming G-C or A-U pairs) to the target RNA (Table 1). Their binding is well predicted to region 115–118 assuming G-U pairing. Probe CUC^L^UC^L^G^L^ is also well predicted to site 114 because the last five nucleotides of the probe are completely complementary to the target RNA. Hybridization in 1 M KCl, 10 mM MgCl_2_ reveals some additional binding sites and overall confirms structure C. The 1 M KCl conditions are less specific and only the new binding to sites 99 and 109 are unambiguous since there are not alternative binding sites for these probes. Higher KCl concentrations apparently make RNA more accessible in internal loop regions, perhaps by competing with Mg^2+^ binding sites.

**Table 1:** Binding of nucleotides 81 to 148 of influenza A NS1 mRNA to isoenergetic probes on microarray.

Binding sites^a^	Modified probe 5′ to 3′^b^	Base pair with G^L^ at 3′ end	RNA binding^c^		ΔG°_37_ predicted (kcal/mol) for complement and alternative binding site^f^	Predicted ΔG°_37_ of modified probe/RNA for complementary binding site^g^
			Buffer I^d^	Buffer II^e^		
91; [127]	UGG^L^GG^L^		M		−8.2; [−6.0]	−9.72
*99*	U^L^CD^L^DG^L^G^L^	C-G^L^		*M*	−*6.9*	−8.68
*102*; *[115*–*118]*	C^L^GD^L^UC^L^G^L^	U-G^L^		*M*	−*7.2; [*−*5.2]*	−9.66
*109*	GC^L^GD^L^AG^L^	C-G^L^		*M*	−*8.1*	−9.87
114	UC^L^UC^L^GG^L^	C-G^L^	S	S	−8.8	−9.35
115	D^L^UC^L^UC^L^G^L^	C-G^L^	S	S	−6.2	−9.17
116; *[100*–*103]*	GD^L^UC^L^UG^L^	G-G^L^	S	M	−6.3; *[*−*4.7]*	−9.02
127; [89–92]; *[96*–*100]*	UD^L^GG^L^GG^L^	U-G^L^	S	M	−7.7; [−7.7]; *[*−*4.7]*	−10.38
128; [90–92]; *[96*–*101]*	U^L^UD^L^GG^L^G^L^	C-G^L^	M	M	−7.2; [−4.5]; *[*−*4.4]*	−8.65
*132*; *138*	CC^L^UC^L^UG^L^	A-G^L^		*S*	−*7.5;* −*6.8*	−10.09
[114]; *[131*–*134]*	CUC^L^UC^L^G^L^		S	S	[−5.8]; *[*−*4.1]*	−11.51
[116]	GG^L^UC^L^UG^L^		S	S	[−5.7]	−12.10
[114/115]	G^L^UC^L^UC^L^G		S	S	[−6.5]	−9.21
[115–118]	GG^L^UC^L^DG^L^		S	S	[−4.6]	−7.73
[115–118]; *[99*–*103]*	GG^L^UC^L^GG^L^		M	M	[−4.6]; *[*−4.8*]*	−7.73

a - Center of binding site, where center is target RNA nucleotide complementary to third nucleotide from 5′-end of probe. Square brackets indicate possible alternative binding sites (if 5 or 6 nucleotides of probe bind) or region of binding (if predicted duplex has less than 5 canonical base pairs). Data in *italics* represent probes that bind in buffer II, but not in buffer I;

b - LNA nucleotides are marked with superscript ^L^, D represents 2,6-diaminopurine, nucleotides without superscript are 2′-O-methyl-nucleotides;

c - S –strong binding, M – medium binding; underline – binding site with no ambiguity;

d - buffer composition is 300 mM KCl, 10 mM MgCl_2_, 10 mM Tris-HCl pH 7.0;

e - buffer composition is 1 M KCl, 10 mM MgCl_2_, 10 mM Tris-HCl pH 7.0;

f - calculated by RNAstructure 4.6 program (for 1 M NaCl, assuming no structure of target RNA and unmodified probe), values correspond to binding sites listed in the first column;

g - calculated for 100 mM NaCl buffer according to published equation [37], [38].

Probes listed underneath the double line have no perfect Watson-Crick match of the first five probe nucleotides to sequences in the target, but bind to target with at least one predicted GU wobble pair or in the case of site 114 have perfect complementarity to the target with only last five nucleotides of the probe. There are no complementary probes for sites: 129–131 and 137 on universal microarrays. Probes complementary to all other sites not listed in table do not bind strongly or moderately.

## Discussion

### Influenza A Nucleotides 81–148 Fold into a Hairpin In Vitro

As seen in Figure 2, the chemical mapping and microarray data support structure C over structure B. In particular, the hairpin loop of structure C is very reactive to chemicals and binds strongly to three microarray probes. In contrast, the reactive nucleotides and probe binding sites are in paired regions of structure B. Evidently, the consensus sequence for this region folds into a hairpin *in vitro* and not a multi-branch loop structure as previously predicted [9]. The results of this study have two important implications: First, in order to improve RNA structure prediction algorithms, the factors that led to multiple previous structure predictions for this region should be considered. Secondly, because functional experiments conducted on this RNA secondary structure assumed a multi-branch loop, new sets of mutations, based on the consensus hairpin structural model, should be designed to further probe the cellular structure and functional role of this domain in host cells.

### Structural Models and The Potential Importance of CA Base Pairs

Defining the functional role of RNA requires knowledge of its likely structure. Based on Mfold [12], [13] and RNAalifold [9], [18] predictions, nucleotides 81–148 of segment 8 were thought to fold into one of two different multi-branch loop structures (Figure 2 and S1A and S1B), one of which may be important for the production of NS1 protein [12]. Both structures are consistent with the known general topology of three-way multi-branch loops [24]. However, the consensus sequence for this region folds into a hairpin structure *in vitro*. The discrepancy between the different structural models may be related to non-canonical base pairs present in some influenza strains. As can be seen from Table C in Figure S1, CA pairs are quite common at positions 106/123, 108/121, and 109/120. Programs, like RNAalifold [18] and Multilign [25], that consider multiple sequences will disfavor structure C because formation of CA pairs is penalized in these algorithms. In the case of Multilign, a base pair is predicted only if all the sequences can form a canonical pair at that position in the developing alignment. Thus, any alignment that contains a CA pair at the positions described above will disfavor prediction of structure C. Similarly, RNAalifold folds the consensus sequence for an alignment and penalizes alignment columns that cannot form a canonical pair above a user-defined threshold. This explains why the hairpin fold (Figure 2C) was not identified in a previous study [9]. However, single-sequence prediction software, including Mfold [13], RNAstructure [15]and RNAfold [26] unanimously predict structure C as the MFE fold for the consensus sequence. In contrast, single sequence folding of the sequence in Figure 2 (panel A) [12] predicts the three-way multi-branch loop because of two variations from the consensus sequence, G123→A creating a CA pair and A138→G changing a potential CA to CG pair (See changes marked on Figure 2B and 2C). CA pairs were found to be between 2 and 45 times more common than other non-canonical base pairs predicted in alignments throughout influenza A coding regions [9]. Until recently it was thought that CA pairs could only form at low pH because the N1 of the adenine needed to be protonated to form a hydrogen bond. However, new studies reveal that adenines within helices can have pKa's shifted to physiological ranges (i.e. pH∼7.0–8.0) [27]. Thus, future investigations into the general occurrence of CA pairs in coding regions and integration of the sequence dependence of energetics for CA pairs may improve predictions using single and multiple sequences. For example, rather than penalizing all non-canonical pairs equally in programs using sequence comparison, it may be better to weight penalties by the individual thermodynamic stabilities of non-canonical pairs. The results for segment 8 nucleotides 81–148 illustrate the importance of comparing predictions from different approaches and testing them experimentally.

### Determining Function for the 41–148 Nucleotide Region

Determining the function for this region will require cell culture and/or *in vivo* assays. The mutations reported by Ilyinskii, et al. [12] that led to NS1 protein down-regulation (NS1mut3841) were designed on expectations that mutation of codon CGA→GCG starting at nucleotide 112 is in a helix that would be disrupted and codon AAA(AAG in consensus) →GCG starting at 121 is in a loop. While chemical mapping shows that the consensus sequence folds into a hairpin *in vitro*, which flips the context of these two codons (Red bars in Figure 2A and 2C), the hairpin structure would also be destabilized by NS1mut3841. In fact, two mutations tested by Ilyinskii, et al. (NS1mut 3841 and NS1mut3540) are expected to perturb both the multi-branch loop and new hairpin model, but only NS1mut3841 caused down-regulation of NS1 protein [12]. Thus, the function of RNA secondary structure in this region is not clear. As previously reported [9], all the codon mutations tested by Ilyinskii, et al. [12] occur in natural sequences except for the alanine introduced by the codon change at residue 121 in NS1mut3841. The combined mutations in NS1mut3841 produce an AXXA amino acid motif that has been reported to cause destruction-box mediated protein degradation [28]. Further mutational studies should focus on making mutations that change RNA secondary structure without changing amino acid sequence and should be conducted on the consensus sequence for which this study provides a confident structural model based on experimental techniques. Such experiments should be able to separate the importance of amino acid effects from those caused by changes in RNA secondary structure. Reverse genetic techniques provide a method for readily introducing mutations into influenza virus, and function can be assayed for a variety of factors including: NS1 protein production, segment 8 splicing, and influenza replication efficiency [29].

An understanding of conserved structure of influenza mRNA can provide new insights into the life cycle of the virus and lead to new therapeutic targets for the treatment of acute influenza infections. For example, if the disruption of a structured domain by site-directed mutagenesis decreases the virulence of a virus (by slowing replication efficiency, etc.), but provides enough viral protein production to elicit an adapted immune response, then these strains may make ideal candidates for live attenuated vaccine development. Because conserved, structured RNA domains are likely fundamental to a wide variety of influenza strains, they could be universal mutation sites for the generation of attenuated viruses.

In conclusion, on the basis of experimental results, the 81–148 region of segment 8 folds into a hairpin structure. However, it should be noted that these results do not preclude the possibility that natural mutations at positions 108, 120, and 123 could favor a reorganization of nucleotides at the top of the structure as is seen in structure A or B, or that this region exists in a structural equilibrium with multiple conformations inside the cell. Other factors present *in vivo*, such as protein binding, can also elicit structural changes. The function of this domain is unclear, but its proximity to the 5′ splice site makes it a possible intronic splicing enhancer/inhibitor [30]. Its location in the segment 8 intron could also serve as a tag to distinguish un-spliced NS1 mRNA from spliced NEP mRNA. Structure C in Figure 2 provides a secondary structure model useful for designing mutation and chemical genetics experiments to test for function and eventually to design potential therapeutics.

## Materials and Methods

### Alignments and Prediction of RNA Secondary Structure

An alignment was created for nucleotides 81 to 148 of the NS1 gene (numbering does not include the UTR) by downloading all available segment 8 sequences (approximately 11,000) from the NCBI Influenza Virus Resource [31]. Sequences were aligned using the MAFFT web server [32]. The 81–148 region was excised from this alignment and the final alignment of 1017 unique sequences was generated by removing all redundant sequences and phylogenetically distinct avian Clade B sequences [33], [34]. Average base pair conservation was calculated from this alignment for each of the three structural models in Figure 2. Structures A and B in Figure 2 are described in Ilyinskii et al. [12] and Moss et al. [9], respectively. Structure C is the minimum free energy structure predicted by RNAstructure [14], [15]. RNAstructure was also used to calculate partition functions for the two sequences in Figure 2 [19].

### Experimental Constructs

DNA oligonucleotide templates were obtained from IDT containing a T7 RNA polymerase site, the consensus sequence for the 81 to 148 nt region, and EcoRI and HindIII restriction sites for cloning (underlined below). The sense DNA construct had the following sequence: 5′-GAATTCTAATACGACTCACTATAGGGTGATGCCCCAT TCCTTGATCGGCTTCGCCGAGATCAGAAGTCCCTAAGAGGAAGAGGCAGCACTCAAGCTT-3′. For chemical mapping studies, an additional nine consensus nucleotides, 5′-TTGGTCTGG-3′, were added at the 3′ end of the consensus sequence, before the HindIII site, to serve as a primer binding site for reverse transcription. The DNA oligonucleotides were annealed and ligated into a digested pUC17 vector. The resulting plasmid was cloned into DH5-alpha cells [Invitrogen]. Plasmid was isolated from cloned cells using a Qiagen mini-prep kit and the sequence was confirmed by sequencing. PCR primers were designed to amplify only the T7 RNA polymerase promoter and the consensus sequence and the resulting product was used for *in vitro* RNA production. RNA was synthesized in vitro using a T7 RNA polymerase kit (Ambion). RNA products were purified on a denaturing 8% PAGE gel and passively eluted overnight in 0.3 M sodium acetate. After ethanol precipitation, the RNA was re-suspended in 0.5 X TE (5 mM Tris, pH 7.5, 0.5 mM EDTA) buffer.

### Chemical Mapping

For all chemical mapping experiments, 10 µL samples were prepared as outlined below. All final concentrations listed are for the final 10 µL mapping samples. Ten pmol of RNA was combined with buffer (final concentration: 10 mM HEPES or potassium borate (CMCT), 100 mM KCl) with a pH of 7.5 (DMS) or 8.0 (NMIA and CMCT). Samples were heated to 95°C for 2 min and then placed on ice for 2 min. MgCl_2_ was added to each sample for a final concentration of 10 mM and the RNA was folded at 37°C for 10 min. As assayed by native gel electrophoresis, this folding protocol produced a single conformation, which runs identically to the single conformation generated with a slow-cooling method [9]. For DMS mapping, 9 µL samples were treated with either 1 µL of 100% ethanol or 1 µL of 1∶30, 1∶60, or 1∶120 dilution of DMS (Sigma-Aldrich) in 100% ethanol for 5 min. NMIA samples were treated with 1 µL of DMSO or 1 µL NMIA (Invitrogen) in DMSO to a final concentration of 12, 6 or 3 mM for a total of 45 min. For CMCT, samples were treated with either 1 µL of water or 1 µL of 50, 25, or 12.5 mg/mL CMCT in water for 5 min. All 10 µL reactions were carried out at 37°C. After incubation, each sample was ethanol precipitated and re-suspended in 10 µL of 0.5 X TE buffer. Primer extension and gel analysis were carried out as described by Wilkinson et al. [35]. Nucleotide positions were identified using dideoxy sequencing ladders. Primer extension was identical to the experimental samples, but 1 µL of A, C, G, or T ddNTP (GE Life Sciences) was added to each reaction.

### Isoenergetic Microarrays

Microarray experiments were carried out as previously described [11], [23]. Isoenergetic microarrays were designed to have modified probes with similar free energies of binding to unstructured RNA [36]–[38]. A universal microarray with 861 different sequences, printed in triplicate, was used. The library of short, modified 2′-O-methyl pentamer and hexamer oligonucleotide probes had LNA and 2,6-diaminopurine substitutions to provide binding independent of sequence of an unstructured RNA. Internal universal negative controls were U, UUUUU, and also spotted buffer. Probes with little complementarity to the 81–148 nucleotide region of the NS1 mRNA also served as negative controls. Microarrays were printed in the European Center of Bioinformatics and Genomics in Poznan, Poland. Prior to hybridization experiments, 5′-end ^32^P labeled RNA was folded as described above. Final folding buffer was 300 mM (or 1 M) KCl, 10 mM Tris-HCl, pH 7.0, and 10 mM MgCl_2_. Microarrays require higher monovalent ion concentrations than used in the chemical mapping experiments to achieve consistent binding [23], [39]. Folded RNA was hybridized to the isoenergetic microarrays for 18 h at 4°C. Microarrays were washed in the same buffer solution, dried by centrifugation and exposed to phosphorimager screen. Obtained data were analyzed using ArrayGauge version 2.1 program as previously described [40], [41]. Intensity of binding was assessed relative to the strongest hybridized probe. Strong and medium binding have ≥1/3 and ≥1/9 of the strongest integrated intensity, respectively. Alternative binding sites were analyzed using RNA-RNA thermodynamics with RNAstructure and prediction of modified probe/RNA free energies [38]. The binding of target RNA to complementary probes is listed in Table 1.

## Supporting Information

Figure S1 **Co-varation analysis of structural models from** **Figure 2** **.** (A, B, and C) Base pair counts in the tables are based on an alignment of 1017 unique sequences. Tables A, B, and C correspond to their respective structures in Figure 2. Base pair conservation and mutations are denoted by different colors on the table, which correspond to the color of the bar between each base pair on the structural models in Figure 2. Canonical pairs are to the left of the vertical line separating the UG and GA columns. A consistent change is a change from a Watson-Crick pair to a GU pair or vice versa. (D) The table at the bottom of the figure gives the amino acid sequence, composition, and percent conservation of each nucleotide position for this region based on the alignment described above.
(TIF)

Figure S2 **Denaturing 8% PAGE analysis for NMIA, DMS and CMCT mapping.** Dideoxy ladders are shown in the first four lanes. NMIA, DMS, and CMCT lanes are marked as being a mock treated control or the amount of reagent used. Strong sites of modification are labeled to the right of the gel. These labels correspond to the site of modification, which is one nucleotide longer than the adjacent cDNA fragment.
(TIF)

Figure S3 **Denaturing 8% PAGE analysis for DMS and CMCT mapping.** Dideoxy ladders are shown in the first four lanes. DMS and CMCT lanes are marked as being a mock treated control or the amount of reagent used. Strong sites of modification are labeled to the right of the gel. These labels correspond to the site of modification, which is one nucleotide longer than the adjacent cDNA fragment.
(TIF)

Figure S4 **Hybridization results for consensus sequence nucleotides 81**–**148 of influenza A NS1 mRNA.** Bar graph represents relative probe binding intensities after hybridization in the following conditions: 300 mM KCl, 10 mM MgCl_2_, 10 mM Tris-HCl, pH 7.0 (Blue) and 1 M KCl, 10 mM MgCl_2_, 10 mM Tris-HCl, pH 7.0 (Red) at 4°C. The average intensities are plotted on the y-axis and the center of probe binding to the target RNA is shown on the x-axis.
(TIF)
