# Supplementary figures and images for: Secondary Structure of a Conserved Domain in the Intron of Influenza A NS1 mRNA
Source: PLoS One. 2013 Sep 2;8(9):e70615. doi: 10.1371/journal.pone.0070615 (PMC3759394; doi:10.1371/journal.pone.0070615)

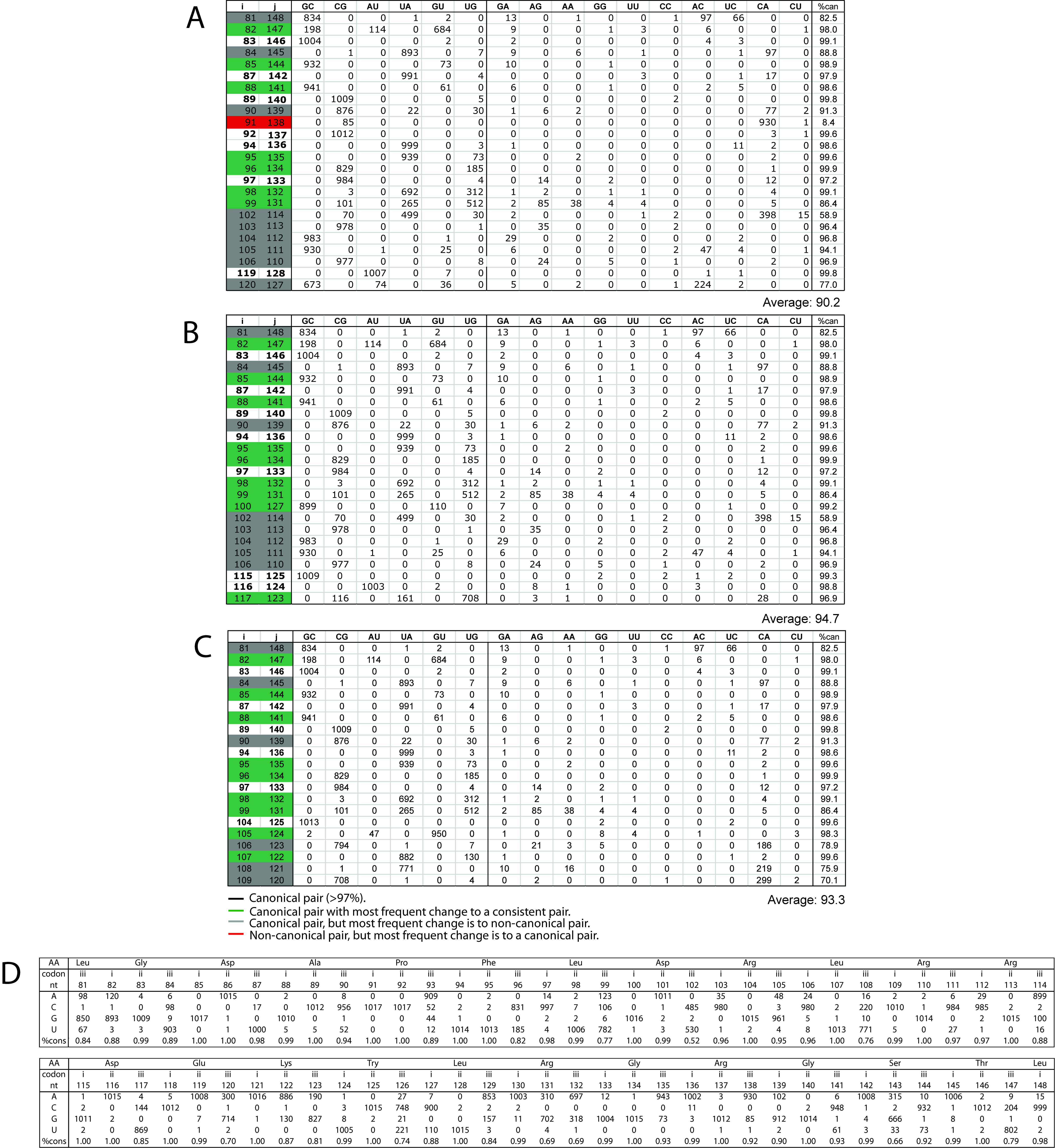

Supplement: Figure S1 — Co-varation analysis of structural models from Figure 2 . (A, B, and C) Base pair counts in the tables are based on an alignment of 1017 unique sequences. Tables A, B, and C correspond to their respective structures in Figure 2. Base pair conservation and mutations are denoted by different colors on the table, which correspond to the color of the bar between each base pair on the structural models in Figure 2. Canonical pairs are to the left of the vertical line separating the UG and GA columns. A consistent change is a change from a Watson-Crick pair to a GU pair or vice versa. (D) The table at the bottom of the figure gives the amino acid sequence, composition, and percent conservation of each nucleotide position for this region based on the alignment described above. (TIF) [file pone.0070615.s001.tif]

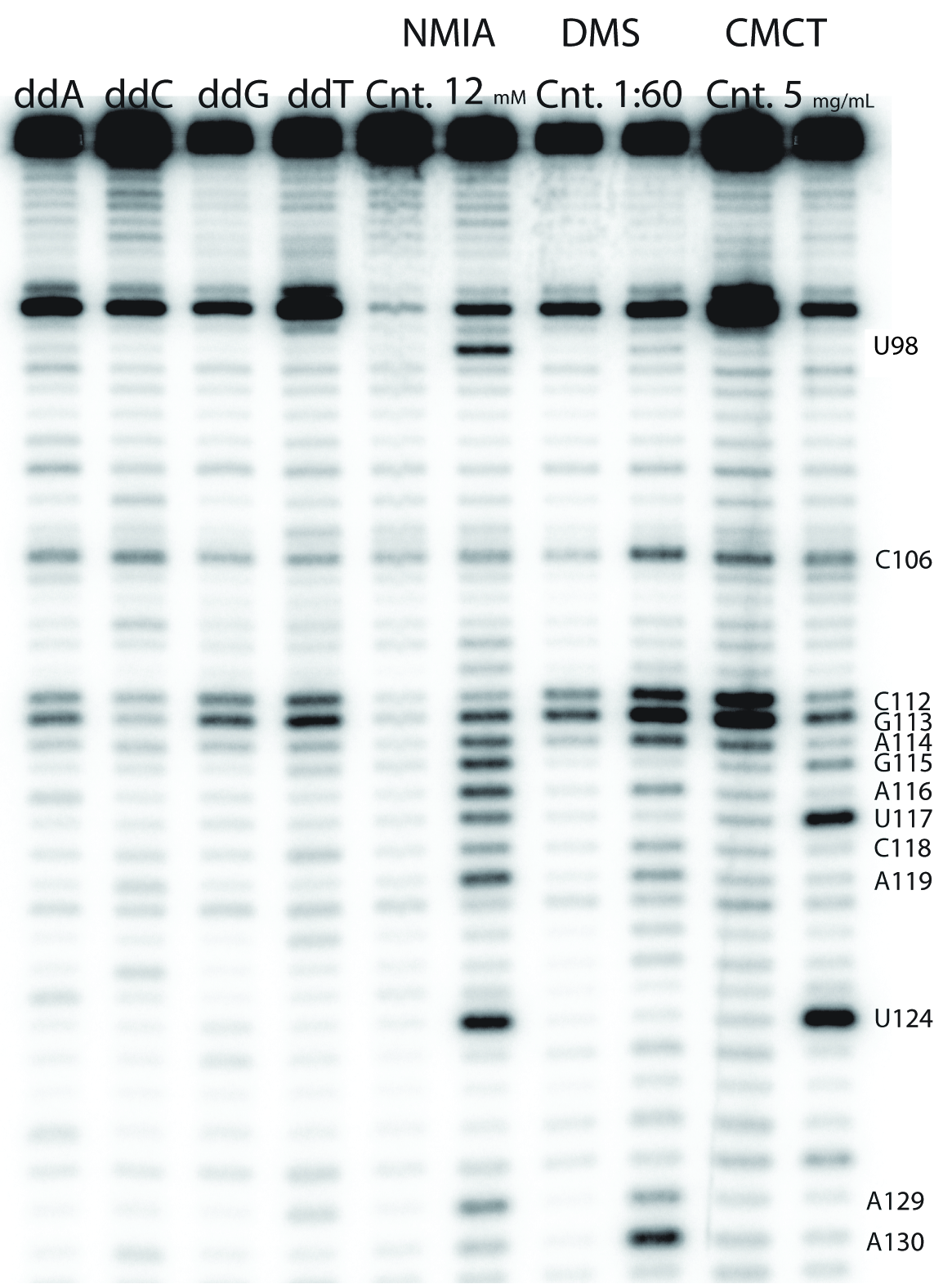

Supplement: Figure S2 — Denaturing 8% PAGE analysis for NMIA, DMS and CMCT mapping. Dideoxy ladders are shown in the first four lanes. NMIA, DMS, and CMCT lanes are marked as being a mock treated control or the amount of reagent used. Strong sites of modification are labeled to the right of the gel. These labels correspond to the site of modification, which is one nucleotide longer than the adjacent cDNA fragment. (TIF) [file pone.0070615.s002.tif]

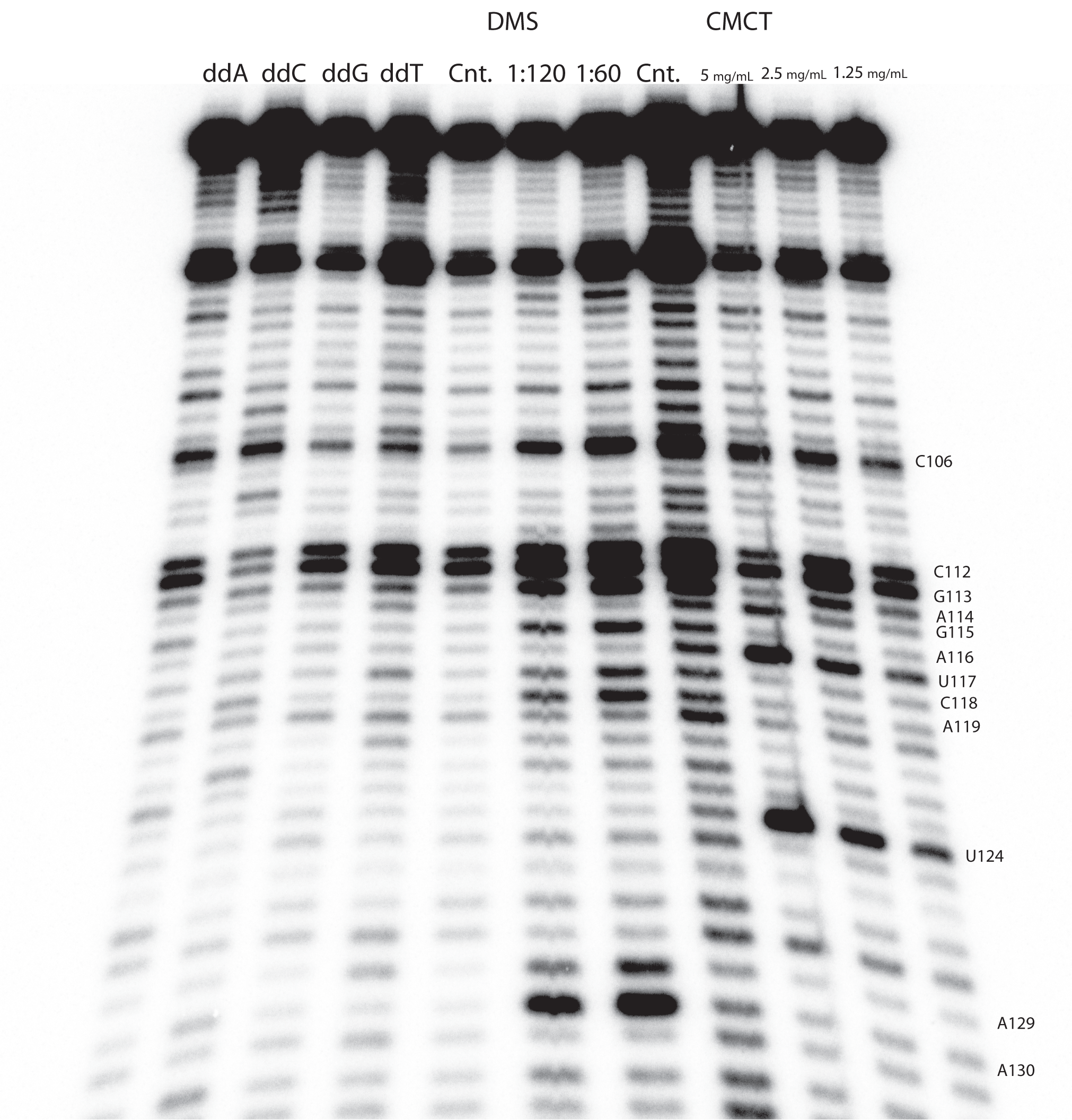

Supplement: Figure S3 — Denaturing 8% PAGE analysis for DMS and CMCT mapping. Dideoxy ladders are shown in the first four lanes. DMS and CMCT lanes are marked as being a mock treated control or the amount of reagent used. Strong sites of modification are labeled to the right of the gel. These labels correspond to the site of modification, which is one nucleotide longer than the adjacent cDNA fragment. (TIF) [file pone.0070615.s003.tif]

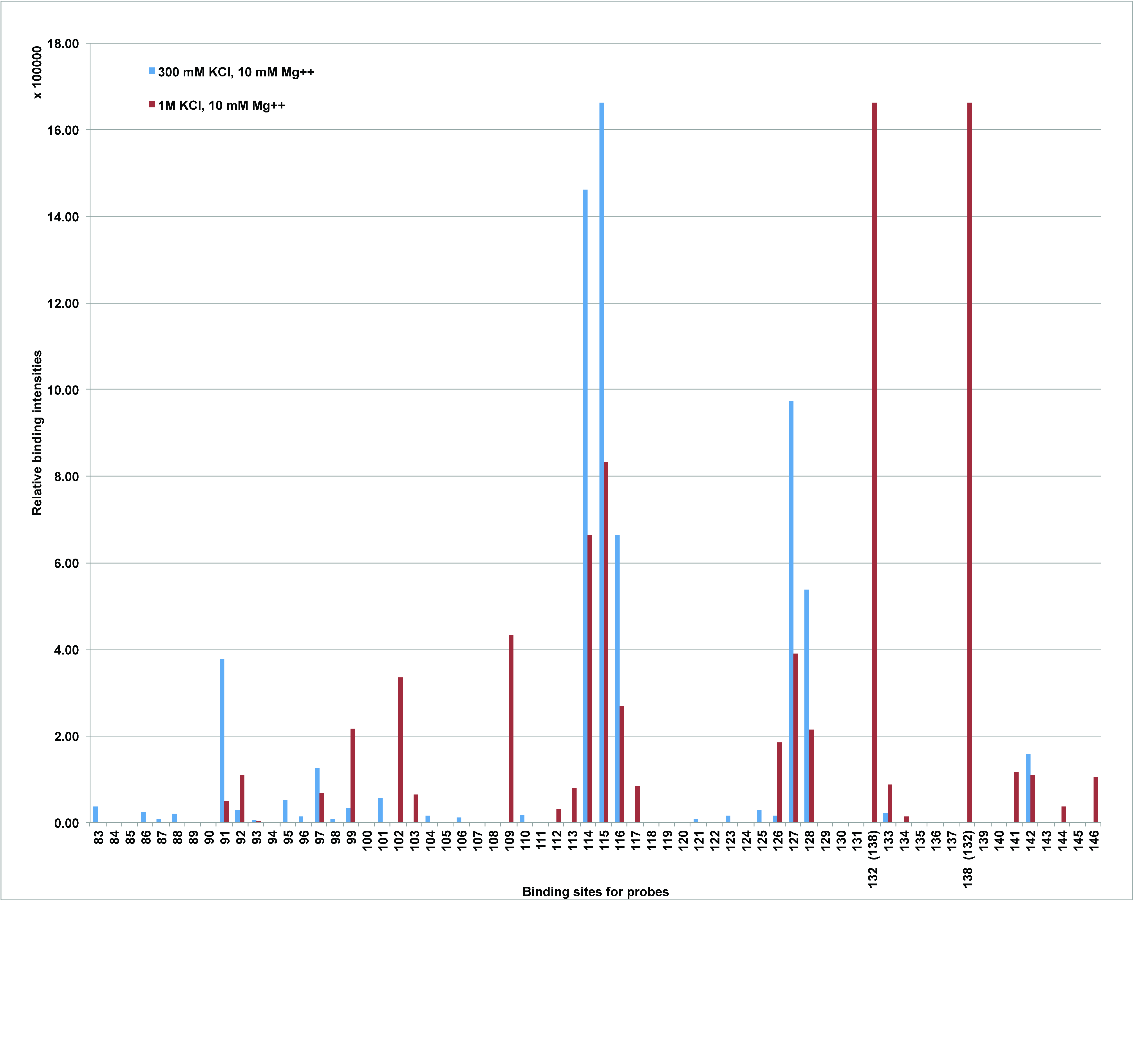

Supplement: Figure S4 — Hybridization results for consensus sequence nucleotides 81–148 of influenza A NS1 mRNA. Bar graph represents relative probe binding intensities after hybridization in the following conditions: 300 mM KCl, 10 mM MgCl2, 10 mM Tris-HCl, pH 7.0 (Blue) and 1 M KCl, 10 mM MgCl2, 10 mM Tris-HCl, pH 7.0 (Red) at 4°C. The average intensities are plotted on the y-axis and the center of probe binding to the target RNA is shown on the x-axis. (TIF) [file pone.0070615.s004.tif]
